# Non-typeable *Haemophilus influenzae *and *Streptococcus pneumoniae *as primary causes of acute otitis media in colombian children: a prospective study

**DOI:** 10.1186/1471-2334-11-4

**Published:** 2011-01-05

**Authors:** Alexandra Sierra, Pio Lopez, Mercedes A Zapata, Beatriz Vanegas, Maria M Castrejon, Rodrigo DeAntonio, William P Hausdorff, Romulo E Colindres

**Affiliations:** 1 Centro de Estudios en Infectologia Pediatrica CEIP, Cali, Colombia; 2 GlaxoSmithKline Biologicals, Panama City, Panama; 3 GlaxoSmithKline Biologicals, Wavre, Belgium; 4 GlaxoSmithKline Biologicals, Rio de Janeiro, Brazil

## Abstract

**Background:**

Acute otitis media (AOM) is one of the most frequently encountered bacterial infections in children aged < 5 years; *Streptococcus pneumoniae *(*S. pneumoniae*) and non-typeable *Haemophilus influenzae *(NTHi) are historically identified as primary AOM causes. Nevertheless, recent data on bacterial pathogens causing AOM in Latin America are limited. This prospective study aimed to identify and characterize bacterial etiology and serotypes of AOM cases including antimicrobial susceptibility in < 5 year old Colombian children.

**Methods:**

From February 2008 to January 2009, children ≥3 months and < 5 years of age presenting with AOM and for whom a middle ear fluid (MEF) sample was available were enrolled in two medical centers in Cali, Colombia. MEF samples were collected either by tympanocentesis procedure or spontaneous otorrhea swab sampling. Bacteria were identified using standard laboratory methods, and antimicrobial resistance testing was performed based on the 2009 Clinical and Laboratory Standards Institute (CLSI) criteria. Most of the cases included in the study were sporadic in nature.

**Results:**

Of the 106 enrolled children, 99 were included in the analysis. Bacteria were cultured from 62/99 (63%) of samples with *S. pneumoniae, H. influenzae, or S. pyogenes*. The most commonly isolated bacteria were *H. influenzae *in 31/99 (31%) and *S. pneumoniae *in 30/99 (30%) of samples. The majority of *H. influenzae *episodes were NTHi (27/31; 87%). 19F was the most frequently isolated pneumococcal serotype (10/30; 33%). Of the 30 *S. pneumoniae *positive samples, 8/30 (27%) were resistant to tetracycline, 5/30 (17%) to erythromycin and 8/30 (27%) had intermediate resistance to penicillin. All *H. influenzae *isolates tested were negative to beta-lactamase.

**Conclusions:**

NTHi and *S. pneumoniae *are the leading causes of AOM in Colombian children. A pneumococcal conjugate vaccine that prevents both pathogens could be useful in maximizing protection against AOM.

## Background

Acute otitis media (AOM) is one of the most frequent bacterial infections in infancy and early childhood worldwide. Though diagnosed in all ages, AOM is relatively common among children between six months to three years of age with approximately 80% of children having had at least one episode of AOM by the time they are three years old [1]. AOM is one of the main causes of childhood morbidity in both developed and developing countries [2]. The estimated annual disease burden of AOM ranges between 8,200,000 and 12,900,000 cases among children less than five years of age in Latin America and the Caribbean [3], compared to that in US children [4] where the estimated number of AOM per year are 10,200,000. In Europe, the high incidence of AOM during childhood has been confirmed, estimated to range from 17,600 to 38,700 episodes per 100,000 person-years in children under five years of age [5].

While both bacteria and/or viruses can cause AOM, the most serious infections are believed to involve bacterial pathogens [6], particularly *Streptococcus pneumoniae *(*S. pneumoniae*) or non-typeable *Haemophilus influenzae *(NTHi), which together account for at least 60-70% of clinical AOM episodes [7]. Other less frequently reported bacterial agents of AOM are *Moraxella catarrhalis *(*M. catarrhalis*), *Streptococcus pyogenes *(*S. pyogenes*) and *Staphylococcus aureus (S. aureus) *[8,9]. Limited studies from Latin America have suggested that *S. pneumoniae *is the most common bacterial pathogen found in middle ear fluid samples from AOM cases [10,11]. However, in recent years, *Haemophilus influenza*e (*H. influenzae*) is increasingly being recognized as a prominent bacterial pathogen in AOM [11,12]. In addition, many of the AOM etiology studies referred to above are centered on treatment failure or persistent AOM patients and not sporadic cases even though the latter represent the vast majority of AOM, and thus, even the global data may not reflect the true etiology of the most common form of AOM [13].

Although some AOM cases resolve spontaneously, the majority receives antibiotic treatment in order to diminish the duration of symptoms and increase the likelihood of resolution [14]; AOM is one of the primary reasons for pediatricians to prescribe antibiotics [15-17]. Constant use of antibiotics over the years has led to an increase in resistance/non-susceptibility to antibiotics of the bacterial pathogens. Some of the antibiotic-resistant serotypes of *S. pneumoniae *are the ones that are most often involved in serious pneumococcal infections [18]. In Latin America, the serotypes 6A, 6B, 9V, 14, 19A, 19F, and 23F are responsible for most of the antibiotic-resistant

*S. pneumoniae *infections that cause invasive disease in children [19]. According to the Surveillance reports of the Regional System for Vaccines (SIREVA), coordinated by the Microbiology Group of the National Institute of Health of Colombia, of the 90 different serotypes of *S. pneumoniae *identified so far, serotype 14 is the most frequently isolated serotype in Colombia for invasive pneumococcal disease [20].

Widespread immunization of children with pneumococcal conjugate vaccines might play a role in reducing AOM episodes. The heptavalent pneumococcal conjugate vaccine (*Prevnar*™/*Prevenar*™, Pfizer/Wyeth, USA; PCV-7) was introduced as public immunization for US children in 2000. In clinical efficacy trials, this vaccine had previously been shown to have 57% efficacy against vaccine-type pneumococcal AOM [21], and overall 7% efficacy against clinical AOM episodes [22]. With the use of antibiotics at high doses and PCV-7, there has been a change in the pneumococcal serotypes responsible for persistent AOM and an increasing predominance of *H. influenzae *[23,24].

In the recently licensed 10-valent pneumococcal/*H. influenzae *protein D conjugate vaccine (*Synflorix*™, GlaxoSmithKline [GSK] Biologicals, Rixensart, Belgium; PHiD-CV) that contains the same serotypes as found in PCV-7 plus the invasive serotypes 1, 5, and 7F, 8 of the 10 serotypes are conjugated to a recombinant form of protein D of non-typeable *H. influenzae*. A randomized, double-blind, controlled study with a prototype 11-valent protein D formulation suggests that PHiD-CV may provide similar protection against pneumococcal AOM as that seen with PCV7 and additional protection (35%) against episodes of AOM caused by NTHi [25]. A 13-valent pneumococcal vaccine formulation (PCV-13) is yet to be licensed in Colombia.

Current data on etiology of AOM in Colombian children are limited and scarce, with one study on Colombian children with AOM conducted over 20 years ago [6]. After the 7-valent pneumococcal conjugate vaccine was introduced in the Colombian private markets in early 2002, it remains important to characterize the bacteria responsible for AOM and to evaluate the potential impact and effectiveness of vaccination with conjugate vaccines against *H. influenzae *and/or pneumococcal AOM. This prospective study aims to identify and characterize bacterial etiology and serotypes including antimicrobial susceptibility of AOM in Colombian children less than five years of age.

## Methods

### Study design and subjects

This is a prospective epidemiological study conducted in routine clinical setting at two medical centers in Cali, Colombia. Children between three months and five years of age, visiting two pediatric clinics for AOM from February 2008 to January 2009, and for whom a middle ear fluid (MEF) sample was available were enrolled in the study.

The study included children visiting the pediatrician with one of the general signs for AOM (otalgia/irritability, conjunctivitis, fever and either Paradise's criteria (bulging, diffused, or localized inflamed tympanic membranes) or spontaneous otorrhea (less than 24 hours). Patients identified for recruitment were either children with a new episode of AOM (less than 72 hours of onset) who had not yet received antibiotics for the episode (untreated group), or children who had a diagnosis of AOM within 48-72 hours prior to study enrolment, and who received antibiotic therapy from a physician but remained symptomatic at the time of study entry (treatment failures). Children who received systemic antibiotic treatment for a disease other than AOM in the 72 hours prior to enrolment and ones receiving antimicrobial prophylaxis for recurrent AOM were excluded. Recurrent AOM was defined as ≥ 3 episodes in the past 6 months or ≥ 4 episodes in the past 12 months. There were no restrictions on antibiotic use following tympanocentesis. All children were treated according to local practices.

The primary endpoints were occurrence of *H. influenzae*, *S. pneumoniae *and other bacterial pathogens isolated from MEF samples. Secondary endpoints were occurrence of *H. influenzae *and *S. pneumoniae *specific serotypes.

Written informed consent was obtained from the parent/guardian of the child before conducting any study-related procedures. Demographic information and child's medical history, case history and general symptoms were collected and a clinical examination was performed.

The study included only sporadic cases once they received attention at the study sites using standard criteria accepted internationally for AOM and using standard methods to detect bacterial agents avoiding selection bias.

### Bacterial identification and characterization

Samples of MEF for all children were collected by an ENT specialist by tympanocentesis or by careful sampling of spontaneous otorrhea for children who had spontaneous/accidental rupture of the tympanic membrane. The latter entailed removing and cleaning the ear canal by deep aspiration of the MEF material through the perforation to minimize contamination and spurious results. For the purposes of this study, only *S. pneumoniae*, NTHi, *S. pyogenes *and *M. catarrhalis *were considered true pathogens [26].

Inoculation of samples was done into Amies transport medium and the sample was plated at the GSK designed laboratory within the first 48 hours at room temperature for further analysis. Standard methodology was employed in order to determine the antimicrobial susceptibility. The cut-offs for susceptibility and resistances are provided in Table 1. The samples were coded with the identification number for the child.

**Table 1:** Ranges for S. pneumoniae and H. influenzae antimicrobial susceptibility ote: of companies theentioned in the results now. 36-47 months age group. ren: Potential implications for pneumococcal con

Antibiotic	Susceptible (S) (μg/mL)	Resistant (R) (μg/mL)
***S. pneumoniae***		

Penicillin	≤ 0.06	≥ 2
Cefotaxime	≤ 0.06	≥ 4
Ceftriaxone	≤ 0.06	≥ 4
Chloramphenicol	≤ 2	≥ 32
Erythromycin	≤ 0.06	≥ 1
Levofloxacin	≤ 0.5	≥ 8
Linezolid	≤ 2	≥ 4
Moxifloxacin	≤ 0.25	≥ 4
Ofloxacin	≤ 1	≥ 8
Tetracycline	≤ 1	≥ 16
Trimethoprim/sulfamethoxazole	≤ 0.5/9.5	≥ 16/304
Vancomycin	≤ 1	≥ 2

***H. influenzae***		

Ampicillin	**≤ **1	> 1
Amoxicillin-clavulanate	**≤ **1	> 1
Cefotaxime	**≤ **0.12	> 0.12
Cefuroxime	**≤ **1	> 2
Cefuroxime axetil	**≤ **0.12	> 1
Levofloxacin	**≤ **1	> 1
Erythromycin	**≤ **0.5	> 16
Tetracycline	**≤ **1	> 2
Chloramphenicol	**≤ **1	> 2
Trimethoprim-sulfamethoxazole	**≤ **0.5	> 1

Source: CLSI 2008 - M100-S19- PAGE 65 and 66

### Statistical analyses

The analysis was performed on the According-To-Protocol (ATP) cohort (evaluable subjects meeting all eligibility criteria, complied with the protocol defined procedures, with no elimination criteria during the study and for whom laboratory results of the middle ear fluid sample were available). A recurrent AOM episode was the third or more episode in the last six months or the fourth or more episode in the last 12 months.

The proportions of AOM caused by *S. pneumoniae*, *H. influenzae *and other bacterial pathogens were calculated with their 95% confidence interval (CI). Distribution analysis of *S. pneumoniae *and *H. influenzae *serotypes by age, by procedure and by pneumococcal vaccination status for AOM cases was performed. Seasonality of AOM cases was also determined.

All statistical analyses were performed using statistical analysis system (SAS) version 9.1 and Microsoft Excel (for graphical purposes).

### Ethics

The study was conducted according to Good Clinical Practice guidelines, the Declaration of Helsinki and the local rules and regulations of the country. All the study-related documents were reviewed and approved by the local Independent Ethics Committee and the study adhered to applicable local guidelines.

## Results

### Demography

During the 12-month period, a total of 106 (103 untreated and 3 treatment failures) children were enrolled; all reporting one episode each. Of these, seven children were eliminated from the ATP cohort: two children (treatment failure) received medication (amoxicillin and cefalexin) forbidden in the protocol, one child (treatment failure) had an onset of signs and symptoms of AOM beyond 72 hours of diagnosis, MEF samples could not be collected from three cases, and one child was enrolled twice for the same episode (elimination at the second episode). All 99 (93%) children included in the ATP analyses were untreated with antibiotics; of these, seven cases were classified as recurrent AOM episodes. One child had a bilateral episode. No treatment failures were included in the ATP cohort since none met the enrolment criteria.

The mean age of children was 28.5 months and 54/99 (55%) were males (Table 2). The highest percentage of episodes was reported among children 12-23 months and 24-35 months of age 27/99 (27% each). No statistically significant differences of pathogen distribution by demographic characteristics were noted. Majority of the episodes were positive for at least one *S. pneumoniae*, *H. influenzae *or *S. pyogenes *among children 3-11 months (9/11, 82%), 12-23 months 17/27 (63%), and 24-35 months (18/27; 67%) of age. *H. influenzae *was isolated in all age groups described for this study, 45% (14/31) of these isolates corresponded to children under 2 years of age and reporting the same number of cases in children 3-11 and 12-23 months (n = 7). On the other hand, *S. pneumoniae *was not isolated in children 48-59 months. Among these isolates, 40% (12/30) occurred in children less than 24 months of age, distributed as 3 isolates in children 3-11 (10%) and 9 in 12-23 months (30%).

**Table 2:** Demographic characteristics with bacterial etiology of episodes by age and gender (ATP cohort)

AOM Episodes Characteristics	Categories	Value or n	%
**Age (months)**			-
**Mean**		28.5	-
**SD**		13.03	-
**Median**		29	-
**Range**		5-55	-

**Age group (months)**			

**0-11^a^**		11	11.1%
	*H. influenzae*	7	-
	*S. pneumoniae*	3	-
	*S. pyogenes*	0	-
	Others/Negative	5	-

**12-23^b^**		27	27.3%
	*H. influenzae*	7	-
	*S. pneumoniae*	9	-
	*S. pyogenes*	1	-
	Others/Negative	11	-

**24-35**		27	27.3%
	*H. influenzae*	5	-
	*S. pneumoniae*	12	-
	*S. pyogenes*	1	-
	Others/Negative	9	-

**36-47^c^**		26	26.3%
	*H. influenzae*	8	-
	*S. pneumoniae*	6	-
	*S. pyogenes*	0	-
	Others/Negative	13	-

**48-59**		8	8.1%
	*H. influenzae*	4	-
	*S. pneumoniae*	0	-
	*S. pyogenes*	0	-
	Others/Negative	4	-

**Gender**			

**Female ^d^**		45	45.5%
	*H. influenzae*	17	-
	*S. pneumoniae*	14	-
	*S. pyogenes*	1	-
	Others/Negative	17	-

**Male ^e^**		54	54.5%
	*H. influenzae*	14	-
	*S. pneumoniae*	16	-
	*S. pyogenes*	1	-
	Others/Negative	25	-

N = 99; number of episodes

n = number of episodes in a given category

% = n/Number of samples with results available * 100

^a^It includes 3 episodes with more than 1 bacterial isolate (*S. pneumoniae *+ *H. influenzae*+others; *S. pneumoniae *+ others; *H. influenzae *+ others)

^b^It includes 1 episode with more than 1 bacterial isolate (*S. pneumoniae *+ others)

^c^It includes 1 episode with more than 1 bacterial isolate (*S. pneumoniae *+ *H. influenzae*)

^d^It includes 3 episodes with more than 1 bacterial isolate (*S. pneumoniae *+ *H. influenzae*+others; *S. pneumoniae *+ others; *H. influenzae *+ others)

^e^It includes 2 episodes with more than 1 bacterial isolate (*S. pneumoniae *+ *H. influenzae*; *S. pneumoniae *+ others)

More females were positive (31/45, 69%) for at least one study bacteria than males (30/54, 56%). No statistically significant differences of pathogen distribution by gender characteristics were identified. Of the seven recurrent cases, four (15%) belonged to the 36-47 months age group.

### Seasonality

**Figure 1:**
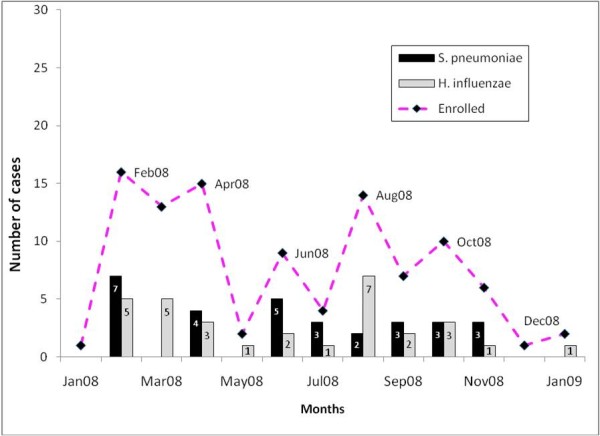
**Seasonal distribution of AOM episodes (ATP cohort)**. The stacked line graph shows the number of episodes enrolled in each month and the simple bar graph shows the number of *S. pneumoniae *and *H. influenzae *isolated each month.

AOM episodes occurred throughout the year; however, the maximum number of cases was enrolled between February and April 2008 with a smaller peak occurring in August 2008 corresponding to the rainy season in Cali. *H. influenzae *was isolated during all months, with higher prevalence in August (n = 7), February (n = 5), and March (n = 5). *S. pneumoniae *was predominantly isolated in February (n = 7) and June (n = 5), but also found throughout the year (Figure 1).

### Sample Collection

Only one MEF sample was collected from 98/99 AOM untreated episodes (one episode had two samples collected, one from each ear). Of the 99 untreated episodes, 84/99 (85%) of samples were collected by tympanocentesis and 16/99 (16%) by spontaneous otorrhea.

### Bacterial etiology

**Figure 2:**
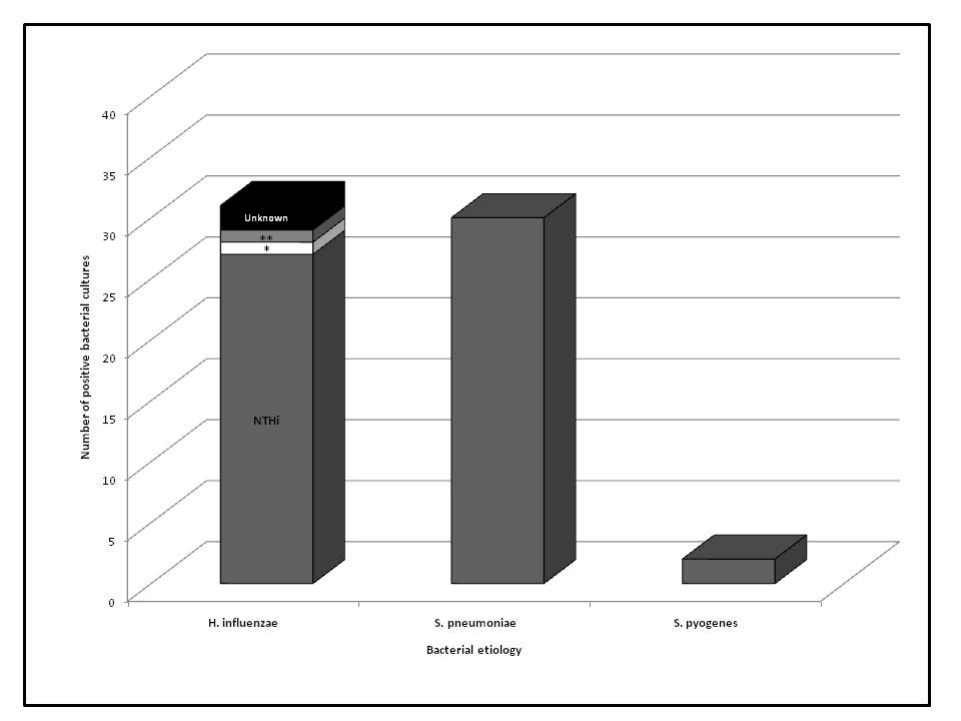
**Etiology of bacteria identified from samples cultured from middle ear fluid**. Number of positive bacterial episodes is represented in the stacked column bar. The bar for *H. influenzae *also includes serotypes of *H. influenzae *positive episodes. * H. influenzae type b ** H. influenzae type f

Pathogenic bacteria were cultured from 62/99 (63%) of episodes with the bacterium: *S. pneumoniae, H. influenzae, or S. pyogenes*. Over 13/16 (81%) of episodes collected by otorrhea swab were bacterial positive, compared to 49/83 (59%) collected by tympanocentesis. Overall, 68 bacterial agents were isolated, corresponding to 56 episodes with a single pathogen and 6 episodes with 2 pathogens. The most commonly isolated bacteria were *H. influenzae *in 31/99 (31%) and *S. pneumoniae *in 30/99 (30%) of episodes, with *S. pyogenes *representing only 2/99 (2%) of cases and other bacteria in 5/99 (5%) cases (Figure 2). Samples collected from two children were positive for both *S. pneumoniae *and *H. influenzae*. No samples tested positive for *M. catarrhalis*. Most of *H. influenzae *and *S. pneumoniae *isolates were taken by tympanocentesis (77% and 83%, respectively). From 83 samples collected by tympanocentesis, 30.1% isolated *S. pneumoniae *and 28.9% isolated *H. influenzae*; and from 16 samples collected by otorrhea, 43.8% corresponded to *H. influenzae *and 31.3% to *S. pneumoniae. H. influenzae *was isolated in 6/7 recurrent AOM episodes, meanwhile *S. pneumoniae *was isolated in only 1/7 recurrent AOM.

At least one dose of the pneumococcal vaccine was received by 29/99 (29%) of children. The percentage of children who received at least one dose of heptavalent pneumococcal conjugate vaccine (PCV-7) was 30% (9/30) include numbers as for H influenza bleow) in children who were culture positive for *S. pneumoniae *(seven fully vaccinated and two partially vaccinated) and 35% who were culture positive for *H. influenzae *(11/31).

### Serotype distribution of *H. influenzae *and *S. pneumoniae*

The majority of *H. influenzae *isolated were non-typeable (27/31; 87%). NTHi was isolated from all *H influenzae *episodes in the 3-11 months, 12-23 months and 48-59 months age groups. In children under two years of age, all isolates corresponded to being non-typeable, meanwhile, 4/17 (24%) encapsulated isolates were identified in the 24-60 months age groups corresponding to f and b serotypes; the remaining 2 isolates were unknown.

**Figure 3:**
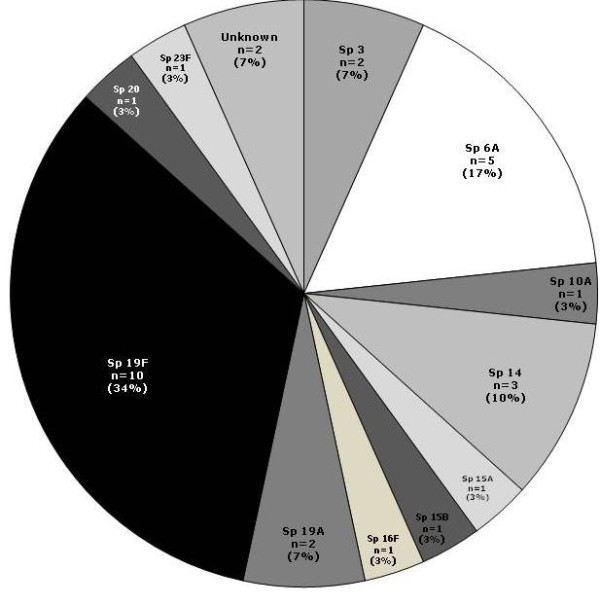
**Serotype distribution of *S. pneumoniae *(N = 30) positive isolates cultured from middle ear fluid samples**. The pie chart shows each serotypes of *S. pneumoniae *with their respective percentage of positive episodes.

Serotype 19F was the most frequently isolated pneumococcal serotype followed by 6A and 14. Serotypes 3 and 19A were the fourth most prevalent (Figure 3). The majority of the serotypes (3, 6A, 14, 16F, 19F, 20, 23F) were isolated in the 12-23 months age group. In fully vaccinated children, serotypes 19F (n = 4), 19A (n = 2) and 6A (n = 1) were isolated. Serotypes 6A and 3 were isolated in the partially vaccinated children.

### Antibacterial susceptibility

**Figure 4:**
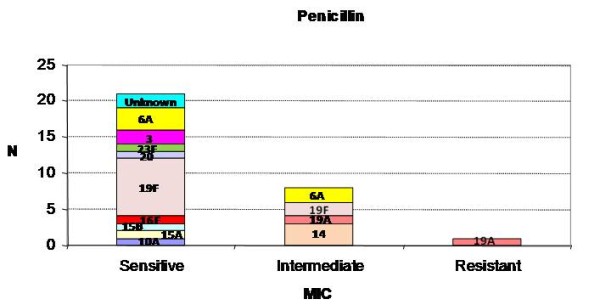
**Antibacterial susceptibility of *S. pneumoniae *positive samples to penicillin (ATP cohort)**. The susceptibility of each *S. pneumoniae *serotype to penicillin for categories sensitive, intermediate and resistant. *Minimum Inhibitory Concentration

The prevalence of sensitive, intermediate and resistant strains for penicillin, among the 30 *S. pneumoniae *positive samples from episodes, were 21/30 (70%), 8/30 (27%) and 1/30 (3%), respectively (Figure 4). The strains resistant to other antibiotics were: 1/30 (3%) to cefotaxime, 5/30 (17%) to erythromycin and 8/30 (27%) to tetracycline and sulfamethoxazole/trimethoprim (Table 3). From *S. pneumoniae *isolates, serotype 19F evidenced antibiotic resistance to tetracycline (50%; 5/10), trimethoprim/sulfamethoxazole (30%; 3/10) and erythromycin (20%; 2/20); serotype 19A was resistant to penicillin, cefotaxime, tetracycline and erythromycin (50%; n = 1/2), respectively.

**Table 3:** Antibacterial susceptibility of samples positive for S. pneumoniae (ATP cohort)

Antibiotics	Serotypes			
	**6A n (%)**	**14 n (%)**	**19A n (%)**	**19F n (%)**

**Penicillin**	3 (60) S 2 (40) I	3 (100) I	1 (50) I 1 (50) R	8 (80) S 2 (20) I

**Cefotaxime**	2 (40) S	1 (33) S 2 (67) I	1 (50) R	7 (70) S 1 (10) I

**Erythromycin**	3 (60) S 2 (40) R	3 (100) S	1 (50) S 1 (50) R	8 (80) S 2 (20) R

**Chloramphenicol**	5 (100) S	3 (100) S	2 (100) S	10 (100) S

**Tetracycline**	5 (100) S	2 (67) S 1 (33) I	1 (50) S,R	5 (50) S 5 (50) R

**Levofloxacin**	5 (100) S	3 (100) S	2 (100) S	10 (100) S

**Trimethoprim/Sulfamethoxazole**	4 (80) S 1 (20) R	3 (100) R	1 (50) S,R	6 (60) S 3 (30) R 1 (10) I

**Other**	5 (100) S	3 (100) S	1 (100) S	9 (100) S

n = number of samples in a given category

(%) = n/Number of samples with results available * 100

S = sensitive; I = intermediate; R = resistant

NT = not tested

Note: None of the serotypes are tested for Amoxicillin/Clavulanate and Cefuroxime; For **serotype 6A**, only 1 sample was sensitive for Azithromycin; For **serotypes 15A and 15B**, only 1 sample was sensitive for each mentioned antibiotic; **Serotype 3 **had 2 samples sensitive for each antibiotic and Not Tested for Cefotaxime; **Serotype 10A **had 1 sample sensitive for all antibiotics; **Serotype 20 **had 1 sample sensitive for all antibiotics, Not Tested for Chloramphenicol and Missing for other; **Serotype 16F and 23F **had 1 sample resistant to Tetracycline, 1 sample Not Tested for Cefotaxime, 1 missing for Other and 1 sample sensitive for other antibiotics

All *H. influenzae *(n = 31) tested with ampicillin were sensitive. From these isolates, 30 were tested for nitrocefin (beta-lactamase test) and reported as negative. All *H. influenzae *samples tested for penicillin (11/11) and for amoxicillin/clavulanate (26/26) were sensitive.

## Discussion

Historically, the major bacteria responsible for most cases of AOM microbiology worldwide does not appear to have changed significantly over time, but the relative prevalence of main causal agents have changed in recent years [9]. In this study, approximately 63% of the samples cultured pathogenic bacteria. This proportion is in line with that seen in another study [27].

*H. influenzae *and *S. pneumoniae *are identified as the main causative agents of AOM in the current study, accounting for 31% and 30% of episodes, respectively. These results are similar to findings from other studies conducted in Latin American countries including Argentina [28], Chile [11], Costa Rica [10,29] and Mexico [30] where the leading causes of bacterial AOM were *S. pneumoniae *and *H. influenzae*. The studies in Argentina, Chile and Mexico looked at sporadic cases; this study shows that major pathogens in sporadic cases are similar to the mentioned studies. Increasing recognition of *H. influenzae *overtaking *S. pneumoniae *as the most frequent cause of AOM has recently been observed [9]. A previous study in the United States (1995-2003) assessed the changes in frequency and pathogens causing AOM since introduction of PCV-7 and reported a significant decrease in *S. pneumoniae *and an increase in *H. influenzae *among MEF isolates suggesting that *H. influenzae *has become the predominant pathogen of AOM since the inclusion of pneumococcal conjugate vaccine in the universal immunization program [13,23]. No significant differences in culture positivity by demographic characteristics and pneumococcal vaccination status were noted in this study with 66% of vaccinated and 61% of unvaccinated children being culture positive for at least one bacteria. However, vaccinated children showed a trend towards non-typeable *H. influenzae *(35%) compared to *S. pneumoniae *(30%).

In this study, *H. influenzae *was detected in all age groups, including during the first two years of life. This finding evidences the major role of this pathogen not only in older subjects as have been previously reported, but also in the younger children where the prominence of *H. influenzae *was not expected. Also, *H. influenzae *was present in most of the samples collected by otorrhea which is opposite from the findings in previous reports where most of otorrhea samples corresponded to *S. pneumoniae*. NTHi was responsible for 27/31 (87%) *H. influenzae *AOM episodes in this study. NTHi has shown to account for more recurrent AOM compared to *S. pneumoniae *and *S. pyogenes *and is associated with a history of recurrent AOM episodes, treatment failure and AOM within two weeks of completing a course of any antibiotic [31], making it a difficult-to-treat pathogen. This was confirmed in this study also where 6/7 recurrent AOM cases were due to NTHi. Similar to previous reports, *M. catarrhalis *and *S. pyogenes *were not major pathogens in this study [28,32,33].

Increase of resistance of *S. pneumoniae *to penicillin and other antibiotics is a concern in Latin America [34]. However, the current study shows limited prevalence of penicillin resistant *S. pneumoniae *but 8/30 (27%) had intermediate resistance to penicillin. Consistent with the findings in Colombia [6], Costa Rica [10] and USA [35], all *H. influenzae *strains tested were beta-lactamase-negative in this study suggesting that both amoxicillin as well as amoxicillin/clavulanate could be considered as an AOM treatment option [36].

From the reported pneumococcal serotypes in this study, 19F was the most frequently isolated pneumococcal serotype in this study, followed by 6A and 14. That four 19F cases were detected in fully vaccinated children appears consistent with the results of the PCV7 AOM efficacy trial conducted in Finland, where the lowest point estimate for all of the PCV7 serotypes was that of 19F, at 25% (95% CI -14 to 51) [21]. In this study, 3 and 19A together represented 13% of the pneumococcal AOM. Serotype 14 has previously been documented to be the most frequent in Colombia [20]. Similar to studies in Costa Rica [10,37] and Chile [11], 19F was the most frequently isolated pneumococcal serotype in this study, followed by 6A and 14. Global data shows the most common pneumococcal serotypes causing AOM are 3, 6A, 6B, 9V, 14, 19A, 19F and 23F [38] indicating that potential coverage for pneumococcal serotypes included in the available formulations ranged from 60-86% [37,39].

The pneumococcal serotypes targeted by PCV-7 and PCV-13 comprise 47% (63% if 6A cross-protection is assumed) and 76%, respectively, of the pneumococci isolated from AOM samples in this study, and thus 14.1% (19.2% assuming 6A cross-protection) and 23.2% of all AOM cases sampled in this study [21,25]. The pneumococcal serotypes targeted by PHiD-CV also comprise 14.1% (19.2% assuming 6A cross-protection) of all AOM cases sampled; in addition, PHiD-CV also targets *H. influenzae *which represents an additional 27% of all AOM cases sampled. It is important to note, however, that neither clinical efficacy nor effectiveness data against AOM are available for either PHiD-CV or PCV-13, and so the magnitude of the clinical impact of each remains undetermined.

There were some limitations to the study. This study is limited by a small sample size which affects the precision of the estimates. In addition, the enrolled children were recruited from private centers which might not be representative of the entire Colombian population. However, we note that both pathogen and serotype distribution are similar to what has been seen in studies elsewhere.

## Conclusions

In summary, *H. influenzae *(NTHi) and *S. pneumoniae *were the most commonly isolated bacteria in the study in all age groups. Vaccination could be an effective strategy in Colombia for preventing AOM disease. Considering the importance of *H. influenzae *as a causative agent of AOM, a conjugate vaccine with efficacy against both *H. influenzae *and *S. pneumoniae *could potentially prevent most bacterial AOM among Colombian children.

## List of abbreviations

AOM: Acute Otitis Media; ATP: According-To-Protocol; CI: Confidence Interval; CLSI: Clinical and Laboratory Standards Institute; GSK: GlaxoSmithKline; *H. influenzae: Haemophilus influenzae;M. catarrhalis: Moraxella catarrhalis;*MEF: Middle Ear Fluid; NTHi: Non-typeable *Haemophilus influenzae;*PCV: Pneumococcal Conjugate Vaccine; PHiD-CV: *H. influenzae *protein D conjugate vaccine; *S. aureus: Staphylococcus aureus;S. pneumoniae: Streptococcus pneumoniae;S. pyogenes: Streptococcus pyogenes*; SAS: Statistical Analysis System; SIREVA: Surveillance reports of the Regional System for Vaccines

## Competing interests

All investigators at study clinical sites were funded though their institutions to do the study.

Maria Mercedes Castrejon, Rodrigo DeAntonio, William P Hausdorff and Romulo E Colindres are employees of GlaxoSmithKline Biologicals.

William P Hausdorff, Romulo E Colindres, Maria Mercedes Castrejon and Rodrigo DeAntonio have stock options.

Pio Lopez received honoraria/paid expert testimony/travel grants from the commercial entity that sponsored the study.

Alexandra Sierra, Beatriz Vanegas and Mercedes Adriana Zapata declare no conflict of interest.

## Authors' contributions

All authors were involved at study conception and design stage and/or acquisition of data and interpretation of data; draft/critical revision of the article and final approval of the manuscript.

## Pre-publication history

The pre-publication history for this paper can be accessed here:

http://www.biomedcentral.com/1471-2334/11/4/prepub
